# RasGAP-Derived Fragment N Increases the Resistance of Beta Cells towards Apoptosis in NOD Mice and Delays the Progression from Mild to Overt Diabetes

**DOI:** 10.1371/journal.pone.0022609

**Published:** 2011-07-25

**Authors:** Natasa Bulat, Evrim Jaccard, Nieves Peltzer, Hadi Khalil, Jiang-Yan Yang, Gilles Dubuis, Christian Widmann

**Affiliations:** Department of Physiology, Biology and Medicine Faculty, University of Lausanne, Lausanne, Switzerland; University of Medicine and Dentistry of New Jersey, United States of America

## Abstract

The caspase-3-generated RasGAP N-terminal fragment (fragment N) inhibits apoptosis in a Ras-PI3K-Akt-dependent manner. Fragment N protects various cell types, including insulin-secreting cells, against different types of stresses. Whether fragment N exerts a protective role during the development of type 1 diabetes is however not known. Non-obese diabetic (NOD) mice represent a well-known model for spontaneous development of type 1 diabetes that shares similarities with the diseases encountered in humans. To assess the role of fragment N in type 1 diabetes development, a transgene encoding fragment N under the control of the rat insulin promoter (RIP) was back-crossed into the NOD background creating the NOD-RIPN strain. Despite a mosaic expression of fragment N in the beta cell population of NOD-RIPN mice, islets isolated from these mice were more resistant to apoptosis than control NOD islets. Islet lymphocytic infiltration and occurrence of a mild increase in glycemia developed with the same kinetics in both strains. However, the period of time separating the mild increase in glycemia and overt diabetes was significantly longer in NOD-RIPN mice compared to the control NOD mice. There was also a significant decrease in the number of apoptotic beta cells *in situ* at 16 weeks of age in the NOD-RIPN mice. Fragment N exerts therefore a protective effect on beta cells within the pro-diabetogenic NOD background and this prevents a fast progression from mild to overt diabetes.

## Introduction

Apoptosis of pancreatic beta cells leads to type 1 diabetes [1], [2] and may contribute to the development of type 2 diabetes [3]. Apoptosis also mediates beta cell loss in islet transplantation both during isolation of the islets [4] and during engraftment [5], [6]. Finding ways of increasing the resistance of beta cells towards apoptotic stimuli would therefore be beneficial in the context of diabetes therapy.

We have characterized in the last few years an amino-terminal RasGAP fragment, called fragment N, that protects various cell types against a series of apoptotic stimuli [7]–[9]. Fragment N is generated by the low caspase-3 activity found in stressed cells and prevents further activation of caspases and apoptosis [8]. In the presence of an apoptotic stimulus, fragment N is further cleaved by caspase-3 and this abrogates its ability to protect cells [9]. It is however possible to prevent this second cleavage by a point mutation in the cleavage site at position 157 [7], [9]. Fragment N mediates its protection by activating the Ras-PI3K-Akt pathway [10]. Despite the fact that Akt can activate the NFkB transcription factor [11]–[13], NFkB stimulation does not occur when Akt is activated by fragment N [10], [14]. This could be beneficial for beta cells because in contrast to what is found in many cell types, sustained activation of NFkB in beta cells induces apoptosis [15]–[18].

We have recently derived a transgenic mouse line in the FVB/N background, called FVBN-RIPN, which expresses a caspase-resistant form of fragment N under the control of the rat insulin promoter (RIP). The presence of fragment N in the beta cells of these mice confer resistance to streptozotocin-induced diabetes [14] and islets isolated from RIPN mice are more resistant to cell death induced by inflammatory cytokines, hyperglycemia, and palmitate [14]. Importantly, the presence of fragment N in beta cells neither impacts on their ability to secrete insulin in response to increased glucose levels nor does it turn on their oncogenic potential [14], [19].

While the ability of fragment N to protect cells against acute apoptotic stimuli has been well established, it is unclear whether this beneficial effect could be observed in the context of a disease that develops on a long-term basis through a progressive increase in apoptosis in a given organ. We addressed this point here by expressing fragment N in the NOD background. The NOD mice, first described in the 1970s [20], represent a useful model of spontaneous development of type 1 diabetes as it shares many similarities with the diseases encountered in humans [21], [22]. Development of diabetes in NOD mice starts by infiltration of immune cells into pancreatic islets. The infiltration is first detected at the periphery of the islets (peri-insulinitis). This occurs around 3–5 weeks of age. Immune cells then invade the islets (insulinitis) so that at 10 weeks of age, 100% of the mice develop severe insulinitis. The tolerance of the infiltrated T cells towards the antigens presented by beta cells is lost in 60–80% of females and 20–30% of males. These mice then experience massive beta cell death and become overtly diabetic. Diabetes development in NOD is driven by T cells because transfer of NOD T cells into irradiated recipients allows the development of the disease, although, interestingly, the T-cell mediated attack only takes place in mice 6 weeks of age and older [23]. The extent of diabetes development in NOD mice is greatly affected by earlier stimulation of the immune system. For example, the incidence of diabetes is highest when the mice are kept in germ-free animal facilities and dramatically decreases when the mice are exposed to foreign antigens encountered in conventional facilities [22]. Modulations of the immune system of young NOD mice can therefore profoundly affect the immune attack on beta cells. This could explain why so many treatments, by compromising the auto-immune attack, prevent the development of diabetes in NOD mice [22]. Consequently, treatments that affect auto-immunity in NOD mice will not provide information on the mechanisms of beta cell destruction that take place in the installation of type 1 diabetes. The NOD model however is very suited to study mechanisms that inhibit or block disease progression once insulinitis has occurred. We present evidence here that fragment N does not affect the extent of the immune attack in NOD mice but that it significantly prolongs their ability to remain in an overt diabetes-free state once their islets have started to be affected by auto-immune cells.

## Materials and Methods

### Ethics Statement

Experiments on the mice were carried out in strict accordance with the Swiss Animal Protection Ordinance (OPAn). The protocol was approved by the Veterinary office of the state of Vaud, Switzerland (Permit Number: 2055).

### Chemicals and antibodies

The Hoechst 33342 dye was purchased from Invitrogen (Basel, Switzerland: catalog number H21492). Recombinant mouse IFNγ was from R&D systems (catalogue number 485-MI). Sodium dodecyl sulfate (SDS) was form Bio-Rad (catalogue number 161-0301). Deoxycholate was from Acros Organics/Thermo Fisher Scientific (catalogue number 302-95-4). Recombinant mouse TNFα was from Enzo Life Sciences (catalogue number ALX-522-009). Dithiothreitol (DTT), Nonidet P-40 (NP40), poly(deoxyinosinic-deoxycytidylic) acid sodium salt (poly dI-dC), HEPES, and recombinant mouse IL-1β were from Sigma-Aldrich (catalogue numbers 43817, I3021, P4929, H3537, and I5271, respectively). Phenylmethylsulfonyl fluoride (PMSF) was from Pierce (catalogue number 206-350-2). The monoclonal antibody specific for the hemagglutinin (HA) tag was purchased as ascites from BabCo (Richmond, CA; catalogue number MMS-101R). This antibody was adsorbed on HeLa cell lysates to decrease non-specific binding [7]. The anti-insulin guinea pig polyclonal IgG antibody (catalogue number 4010-01) was from Linco Research Inc, (St. Charles, MO USA). The anti-CD3 antibody directed at amino acids 156–168 of the human CD3ε chain was from Abcam (catalogue number ab5690). The polyclonal rabbit anti-phospho Akt (serine 473) antibody was from Cell Signaling (catalogue number 9271). The rabbit polyclonal non-phospho-specific anti-Akt 1/2/3 antibody (H-136) was from Santa-Cruz (catalogue number SC-8312). The HRP conjugated anti-rabbit polyclonal antibody was from Jackson ImmunoResearch (catalogue number 211-035-109). The FITC-conjugated goat anti-guinea pig IgG polyclonal antibody (catalogue number 106-095-003), Cy3-conjugated goat anti guinea pig (catalogue number 106-165-003) and the Cy3-conjugated goat anti-mouse IgG polyclonal antibody (catalogue number 115-165-003) were from Jackson ImmunoResearch Europe (Suffolk, UK).

### Buffers

Phosphate buffer saline (PBS): 116 mM NaCl, 10.4 mM Na_2_HPO_4_, 3.2 mM KH_2_PO_4_ (pH 7.25).

Buffer A for nuclear extract preparation: 10 mM HEPES pH 7.9, 1.5 mM MgCl_2_, 10 mM KCl, 0.1 mM EDTA, 0.1 mM EGTA, 0.5 mM DTT, 0.2 mM PMSF, and 0.5% NP40.

Buffer C for nuclear extract preparation: 20 mM HEPES pH 7.9, 420 mM NaCl, 1.5 mM MgCl_2_, 1 mM EDTA, 1 mM EGTA, 1 mM DTT, 10% glycerol, 1 mM PMSF.

Buffer R for EMSA: 10 mM HEPES (pH 7.9), 25 mM KCl, 0.25 mM DTT, 0.1% NP40, 10% glycerol, 1.5 mM MgCl_2_, and 0.1 µg/µl poly dI-dC.

RIPA buffer: 50 mM Tris-HCl (pH 8), 150 mM NaCl, 0.5% deoxycholate, 1% NP40, 0.1% SDS, 1 mM PMSF.

### Cell culture

The rat insulinoma INS1 cell line was cultured at 37°C and 5% CO_2_ in RPMI-1640 (GIBCO; catalogue number 61870) containing 10% fetal bovine serum (GIBCO; catalogue number 10270-106; lot n°41Q6001K), 1 mM sodium pyruvate (Sigma-Aldrich; catalogue number S8636), and 50 µM beta-mercaptoethanol (GIBCO; catalogue number 31350-010; lot n°493563). Isolated islets were maintained in RPMI-1640 medium containing 10% decomplemented fetal bovine serum, 10 mM HEPES, 2 mM glutamine (Sigma-Aldrich; catalog number G-7513), 1 mM sodium pyruvate (Sigma-Aldrich; catalog number S-8636) and penicillin (100 units/ml)/streptomycin (100 µg/ml) (Invitrogen; catalogue number 15140) at 37°C and 5% CO_2_.

### Plasmids

The backbone vector pcDNA3 is from Invitrogen. Myr-mAkt1-HA.cmv (#249), which encodes a constitutively active form of Akt that bears a Src myristoylation sequence at its N-terminus and an HA tag at its C-terminus, was described earlier under the name myr-Akt.cmv [10]. Plasmid hIkB alpha delta N2.cmv (#11) encodes the human IκBα protein with the ΔN2 deletion (i.e. amino acid 3–71); this constructs cannot be phosphorylated by IκB kinases and degraded by the proteasome and therefore functions as an inhibitor of NFκB. It has been described before under the name of IκBαΔN2 [10]. HA-hRasGAP 1–455 (D157A).dn3 (#352) encodes an HA-tagged version of the uncleavable form of human fragment N (described in [7] as N-D157A.dn3). prLUC (#49) is an NFκB reporter plasmid. It contains two NFkB responsive elements (AGGGGACTTTCCGA) and a minimal cFos promoter (nucleotides 498–662 of the mouse cFos gene; locus MMCFOS) upstream of the firefly luciferase. The pEGFP-C1 plasmid encoding the green fluorescent protein (GFP) is from Clonetech.

### Cell transfection

INS1 cells were transfected using lipofectamine 2000 (Invitrogen) with 0.5 µg of a GFP-expression plasmid (to label the transfected cells) and 0.5 µg of the prLUC NFkB-reporter plasmid together with 1 µg of the plasmids encoding the constructs indicated in Figure 2C. The amount of DNA used in the transfection was kept constant to 4 µg by the addition of appropriate amounts of an empty vector (pcDNA3).

### NFκB reporter assay

NFκB activity was measured using a luciferase reporter assay, as described previously [24].

### Transgenic lines

NOD/ShiLtJ-Tg(RIP::N)1Wid mice (abbreviated NOD-RIPN) were obtained by backcrossing the RIPN transgene from FVB/N-Tg(RIP::N)1Wid mice (corresponding to founder 1 in reference [14]) into the NOD background using NOD/ShiLtJ mice (stock n° 001976; The Jackson Laboratory, Bar Harbor, Maine, USA). During the first 5 generations, the progeny containing the highest percentage of NOD genome was selected for further backcrossing. The percentage of recipient genome was determined using the GenoMouse service of Elchrom Scientific (Cham, Switzerland) using 96 polymorphic microsatellite markers. After the fifth generation, the percentage of NOD markers in the selected progeny reached 98.9%. The back-crossing was further pursued for two additional generations at which time the NOD-RIPN mice were used in the described experiments.

### PCR

DNA was isolated from tail or ear biopsies using the two-step extraction “hotSHOT” method [25]. The presence of the transgene was detected by PCR amplification of the isolated DNA, as described previously [14].

### Nuclear extract preparation

Nuclear extracts from islets were prepared as follows: 800 to 1'000 islets were collected in 1.5 ml Eppendorf tubes, washed twice with 800 µl of cold PBS (centrifuged at 2'300 g for 5 minutes in between each wash). The pellet was resuspended in 400 µl ice-cold buffer A in which protease inhibitors (Roche; catalogue number 04693132001) and phosphatase inhibitors (phosSTOP from Roche; catalogue number 04906845001) were freshly added. The islet cells were then incubated on ice for 15 minutes and vortexed 3 times for 15 seconds during the incubation period. After centrifugation for 2 minutes at 16'000 g, the pellets containing the nuclei were resuspended in 35 µl of buffer C in which the same protease inhibitors and phosphatase inhibitors as used in buffer A were freshly added. The samples were then sonicated three times for 5 seconds at an amplitude of 60% using a Vibracell 75186 sonicator (Thermo Fisher Scientific; catalogue number W75186). The samples were then incubated on ice for 20 minutes (the tubes were vortexed 15 seconds three times during this incubation period). Finally, the samples were spun 5 minutes at 16'000 g in an Eppendorf centrifuge and the supernatant stored at −80°C until used.

### Electro-mobility shift assay (EMSA)

The oligonucleotides bearing the NF-κB binding elements correspond to sequence AGC TTC AGA GGG GAC TTT CCG AGA GGA GCT and CCT CTC GGA AAG TCC CCT CTG AAG CTA GCT. Fourty µM of these oligonucleotides were annealed overnight at room temperature in 50 µl of hybridization buffer (2 mM Tris pH 7.4, 1 mM MgCl_2_, 5 mM NaCl). The sticky ends of the annealed double stranded probes were filled using the Klenow fragment of DNA polymerase I (Roche; catalogue number 110 08 409 001) in the presence of deoxycytosine (α-32P)-triphosphate (Hartmann Analytic GmbH; catalogue number SRP-205-123349). The double stranded radio-labeled probes were purified using the Qiagen QIAquick Nucleotide removal kit (catalogue number 28304). Five µg of nuclear proteins were mixed in 20 µl buffer R and incubated with approximately 120 fmoles of the double-stranded labeled oligonucleotides (NF-κB probe) for 20 min on ice. Samples were loaded onto a 6% non-denaturing polyacrylamide gel with 0.5× Tris-borate-EDTA buffer (0.5 mM EDTA, 22 mM boric acid, 22 mM Tris-base). The gels were fixed in a solution of 10% acetic acid and 10% methanol, dried, and exposed to Hyperfilm-MP (Kodak).

### Blood glucose measurement and diabetes incidence measurement

Starting from five weeks of age, NOD and NOD-RIPN mice were subjected to glycaemia measurement once a week. Glycaemia was measured with the Accu-Check Compact plus system (Roche, Mannheim, Germany) using a single drop of blood taken from the tail vein. Mice were considered overtly diabetic if their blood glucose levels were over 20 mM in two consecutive measurements. This was a criterion for euthanasia. If no diabetes developed, the mice were killed at 44 weeks of age.

### Islet isolation

Islets were isolated as described previously [19]. For the experiment described in Figure 2A, the islets were cultured in complete culture medium (see cell culture section) for 22 hours and then maintained in HBSS (GIBCO-Invitrogen; catalogue number 240 20-091) complemented with 25 mM HEPES, 0.1% BSA and 2.8 mM glucose for 2 additional hours. The islets were then washed twice in PBS prior to lysis.

### Western Blot

**Figure 1:**
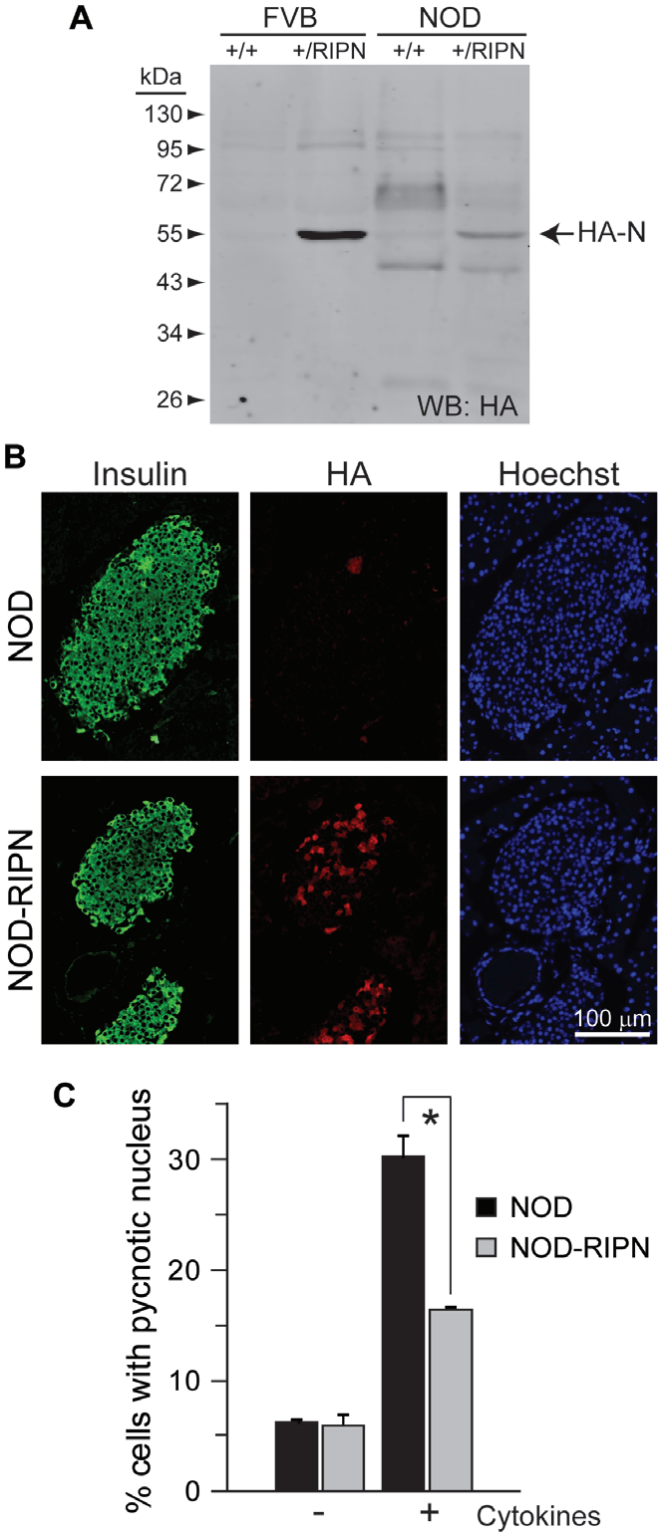
Expression levels and functionality of the RIPN transgene in NOD mice. **A.** Western blot analysis of lysates from islets isolated from ten week-old female mice. The presence of fragment N was assessed using an anti-HA antibody. **B.** Immunohistochemistry analysis of paraffin sections of five week-old female mice. Sections were stained using anti-insulin and anti-HA antibodies. Nuclei were stained with Hoechst 33342. **C.** Freshly isolated islets from 5 week-old females were incubated or not with inflammatory cytokines (1,000 units/ml TNFα, 1,000 units/ml interleukin-1β, and 50 units/ml interferon-γ) during 24 hours. The islets were then stained with Hoechst 33342 and apoptosis was scored.

**Figure 2:**
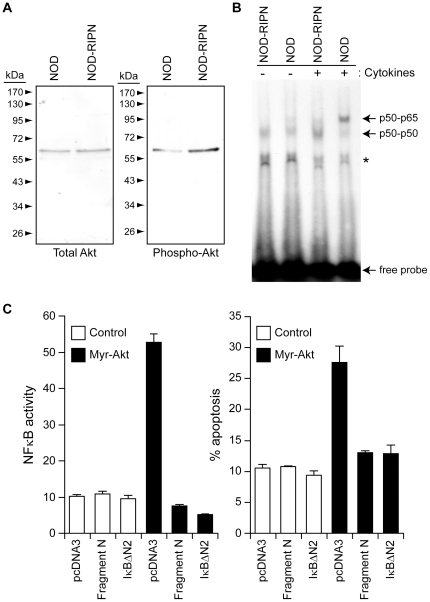
Signals modulated by fragment N. **A.** The extent of Akt activation was assessed by Western blot analysis of 10 µg of lysates from islets isolated from 5 week-old female mice using an antibody recognizing the phosphorylated form of Akt. Total levels of Akt were also determined using a non-phospho-specific anti-Akt antibody. **B.** Islets isolated from NOD and NOD-RIPN mice were stimulated or not for 30 minutes with inflammatory cytokines (1,000 units/ml tumor necrosis factor-α, 1,000 units/ml interleukin-1β, and 50 units/ml interferon-γ). The ability of nuclear proteins to interact with an NFκB-binding element-bearing radioactive probe was then monitored by EMSA as described in the methods. The locations of p65-p50 and p50-p50 complexes are indicated. The asterisk denotes a nonspecific band. **C.** INS1 cells were transfected with an empty vector (pcDNA3), a plasmid encoding the myristoylated active form of Akt (myr-Akt), a plasmid encoding a super NFκB repressor (IkBαΔN2), or a plasmid encoding fragment N in the indicated combinations, together with a NFκB-reporter luciferase construct and a GFP-encoding plasmid (to label the transfected cells). One day after, transfection, the cells were lysed to assess NFκB activity (left panel). Alternatively, apoptosis was scored in the transfected cells (right panel).

After isolation, islets were lysed on ice in mono Q-c buffer [7] supplemented with one tablet of EDTA-free protease inhibitor per 50 ml (Figure 1A) or in RIPA buffer supplemented with one tablet of phosphatase inhibitors (Roche; catalogue number 04 906 845 001) per 50 ml (Figure 2A). Protein concentration was measured by Bradford assay using BSA as a standard. Lysates were mixed with sample buffer {62.4 mM Tris-HCl (pH 6.8), 10% glycerol, 5% β-mercaptoethanol (v∶v), 2% sodium dodecyl sulfate (w∶v), and 0.01% bromophenol blue} before loading on SDS-PAGE gels. Western blotting was performed and quantitated as described previously [26].

### Preparation of tissue section and immunohistochemistry

Histology sections were prepared as described previously [14]. Briefly, five, ten, and sixteen week-old mice were anesthetized using 100 mg/kg pentobarbital injected i.p. (50 mg/ml, Lausanne University Hospital, Switzerland, lot n° P08FA) and perfused with 150 ml of PBS containing 4% paraformaldehyde (PFA; Acros Organics, catalog number 416780010). The isolated pancreata were further fixed in PBS/4% PFA for 2 hours. After this fixation step, pancreata were stored in PBS/4% formol solution and embedded in paraffin. Eight µm sections were deparaffinized in toluene (Carlo Erba, Milan, Italy, catalog number 488555,) and rehydrated using graded alcohol and distilled water. Antigen retrieval was performed by immersing sections in sodium citrate buffer (10 mM sodium citrate pH 6), followed by heating in a microwave for 20 minutes (8 min at 800 Watts and 12 minutes at 400 Watts). Sections were cooled down to room temperature, blocked using a 50 mM Tris-HCl pH 7.6, 0.5% tween 20, 0.2% BSA solution. The primary anti-CD3 antibody was diluted 1/200, whereas the primary anti-insulin and anti-HA antibodies were diluted 1∶100, in 50 mM Tris-HCl pH 7.6, 0.5% tween 20, 0.2% BSA and incubated with the slides for 1 hour. Slides were washed 2 times 10 min in 50 mM Tris-HCl pH 7.6, 0.5% tween 20. The fluorochrome-conjugated secondary antibody, diluted in 50 mM Tris-HCl pH 7.6, 0.5% tween 20, 0.2% BSA, was incubated with the slides for another hour in the dark. Slides were then extensively washed (at least 6 times with one overnight washing step). The nuclei in the sections were then stained with 10 µg/ml Hoechst 33342. Finally, the slides were mounted in Vectashield mounting medium (Vector laboratories Inc, Burlingame, CA USA).

### Immunohistochemistry tyramide signal amplification

Eight µm sections were processed as described above until the antigen retrieval step. The endogenous peroxidase enzymes in the paraffin sections were then inhibited with a 3% H_2_O_2_ solution for 10 minutes. Slides were washed 3 times in PBS, 0.05% tween 20 and then blocked in PBS, 0.05% tween 20, 0.2% BSA. The primary anti-CD3 antibody was diluted 1/200 in the blocking solution and incubated with the slides for 1 hour. Slides were washed 3 times 10 min in PBS, 0.05% tween 20. The HRP-coupled secondary antibody, diluted 1/1000 in PBS, 0.05% tween 20, 0.2% BSA, was incubated with the slides for another hour in the dark. Slides were then washed 3 times in PBS, 0.05% tween 20 and incubated for 10 minutes in the dark with the Cy3 amplification reagent diluted 1/50 in 1× Plus Amplification Diluent according to the manufacturer instructions (TSATM Plus Cyanine 3 System; Perkin Elmer NEL744001KT). Slides were extensively washed in PBS, 0.05% tween 20 (4 times with one overnight washing step). The nuclei in the sections were then stained with 10 µg/ml Hoechst 33342. Finally, the slides were mounted in Mowiol (Fluka; catalogue number 81381) at a concentration of 0.1 mg/ml in a solution made of 20% glycerol and 0.1% DABCO (diazobiciclo-octane; Fluka; catalogue number 33480).

### Quantitation of CD3-positive cells within islets

Quantitation was performed with the NIS-Elements AR program (Nikon) using the taxonomy tool of the “annotations and measurements” menu. The total number of cells within an islet (including infiltrating immune cells) was determined by scoring the number of nuclei (stained with the Hoechst dye). The number of T cells within islets was determined by scoring the number of cells stained with the Cy3-labelled anti-CD3 antibody.

### Whole section area quantitation

Sections stained as described in the previous sections were scanned using an automated Nikon Eclipse 90i microscope equipped with a 20× objective and piloted with the NIS-Elements Basic Research software (Nikon Instruments INC. Melville, USA). The pictures were then converted to grayscale using Adobe Photoshop Elements 5.0. They were then analyzed using the ImageJ software (http://rsbweb.nih.gov/ij/) as follows: the area of the whole pancreas was determined by setting a threshold to remove the surface not occupied by tissues. The area of the insulin positive cells was measured manually using the polygonal tool of the software. The number of insulin positive cells within islets was assessed manually by counting nuclei of the insulin positive cells. The surface of individual insulin-containing cells was calculated by dividing the area occupied by insulin-positive cells by the number of insulin-positive cells in a given pancreatic section.

### Apoptosis scoring

Apoptosis on histological slides was assessed by TUNEL assay (DeadEnd Fluorometric TUNEL system, Promega Switzerland catalog number G3250) on islet paraffin sections as per the manufacturer's protocol. Apoptosis *ex vivo* or *in vitro* was assessed by scoring the number of cells with pycnotic nuclei after Hoechst 33342 staining [19]. In the case of INS1 cells, only the transfected cells (i.e. expressing GFP) were scored.

### Infiltration scoring

Lymphocytic infiltration was scored based on Hoechst 33342 staining of islet paraffin sections. Cells with small nuclei were considered of haematopoietic origin. Infiltration scores were determined as follows: 0, absence of small nuclei around or near the islet, 1, presence of small nuclei around the islet but no or very limited infiltration, 2, up to one third of the islet infiltrated with cells with small nuclei, 3, between one third to two thirds of the islet infiltrated with cells with small nuclei; and 4, more than two thirds of the islet infiltrated with cells with small nuclei.

### Statistics

**Figure 3:**
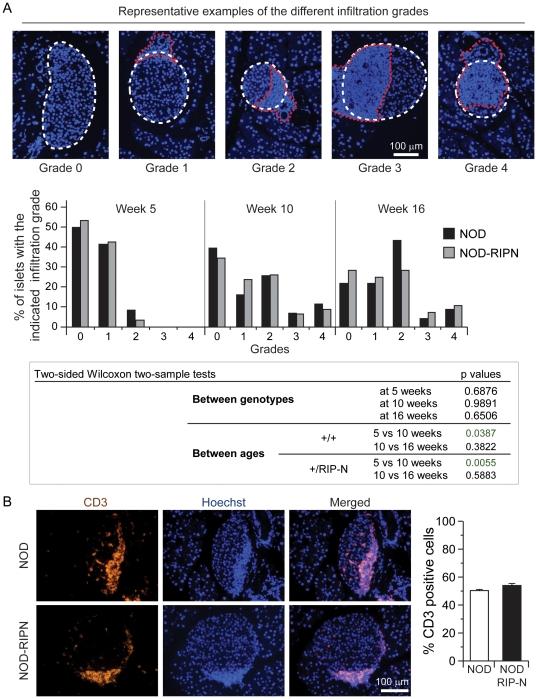
Lymphocytic infiltration. **A.** Paraffin sections of female mice of the indicated ages were stained with Hoechst 33342. Infiltration was scored as described in the methods. The upper images depict representative examples of the different infiltration grades (the white dotted lines delineate the islets and the red dotted lines encircle the lymphocytic infiltration). **B.** Paraffin sections of infiltration grade 2 islets of 10 weeks old NOD and NOD-RIPN mice were stained with an anti-CD3 specific antibody (orange). The nuclei were stained with Hoechst 33342 (blue). Quantitation of CD3-positive cells relative to the total number of islet cells is shown on the right. Note that T cells (i.e. CD3-positive cells) are smaller than islet cells. Hence T cells making about 50% of the total number of cells within islets occupy an islet area that is less than half.

**Figure 4:**
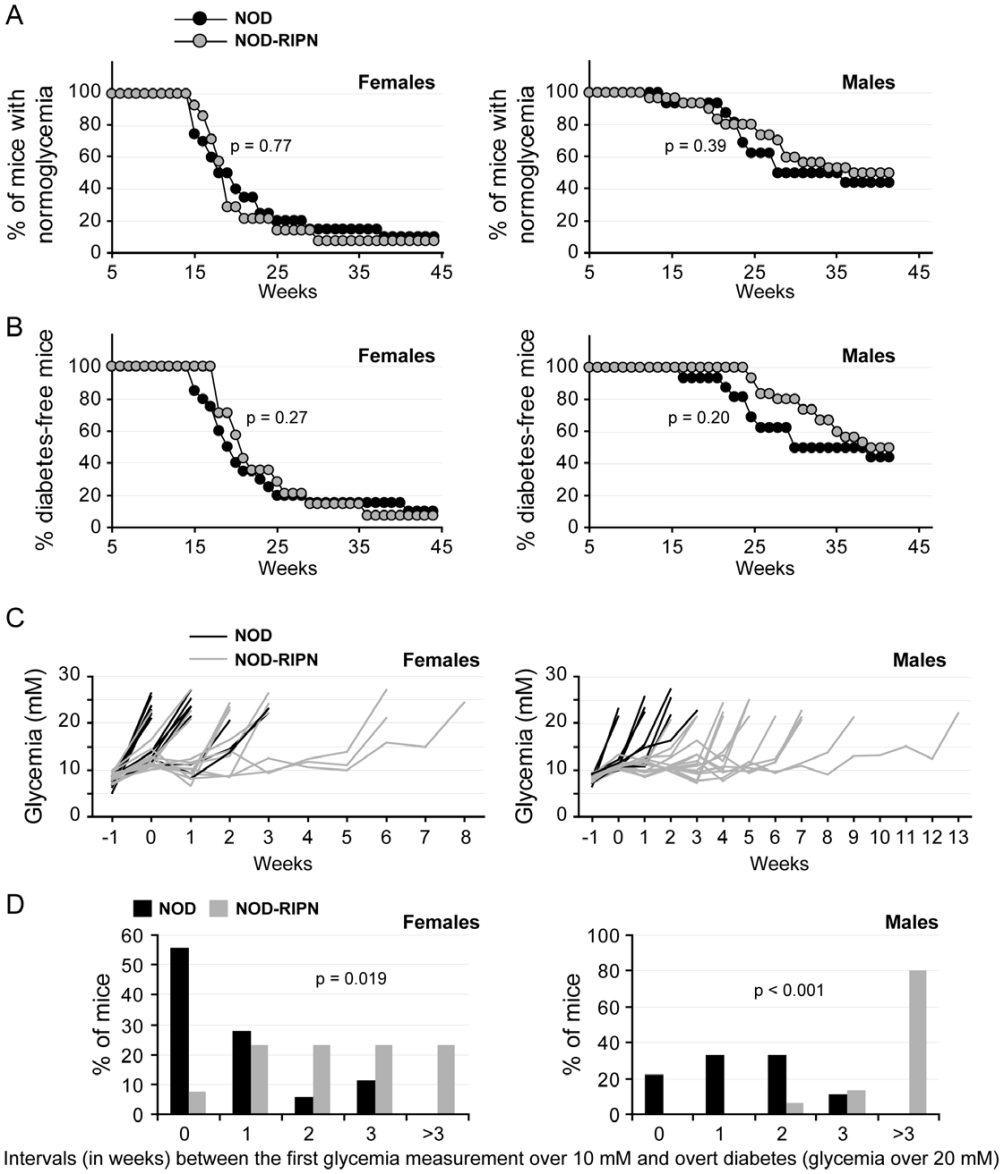
Diabetes development. The glycaemia of non-fasted mice (20 NOD females, 14 NOD-RIPN females, 16 NOD males, and 30 NOD-RIPN males) was measured once a week. Mice with glycaemia under 10 mM were considered normo-glycaemic and those with a glycaemia over 20 mM were considered diabetic. **A.** Normo-glycaemia curves. **B.** Diabetes-free curves. **C.** Glycaemia of individual mouse preceding the development of overt diabetes. Time 0 corresponds to the time when the mice had a glycaemia over 10 mM for the first time. **D.** Panel C was used to determine the percentage of mice that had glycaemia over 10 mM for the indicated number of weeks before becoming overtly diabetic.

The SAS/STAT v9.1 software (SAS Institute Inc., Cary, NC, USA) was used to perform the statistical analyses. Two-sided Wilcoxon two-sample tests were used in Figure 3. For figures 4A and B, Wilcoxon tests of equality over strata (life-test procedure) were performed. Fisher exact tests were used to analyze the data shown in Figures 4D. The remaining data were analyzed using student t tests with Bonferonni corrections. Asterisks and “NS” in the figures indicate significant differences and no significant differences, respectively. Unless otherwise stated, results are derived from triplicate determinations (from three independent experiments) or three animals per condition and are presented as means ±95% confidence intervals (95% CI).

## Results

### Expression of fragment N in pancreatic beta cells of NOD mice

The RIPN transgene encoding the HA-tagged form of the D157A caspase-resistance fragment N form under the control of the rat insulin promoter (RIP) [14] was backcrossed into the NOD background (see materials and methods), generating NOD-RIPN mice. Table 1 indicates that the size of islets and islet cells, the number of insulin-positive cells per islet, and the number of islets per section were not affected by the RIPN transgene. Figure 1A shows that fragment N is expressed in islets isolated from NOD-RIPN albeit to lower levels compared to mice expressing the transgene in the FVBN background (FVBN-RIPN mice). This weaker expression likely results from a mosaic expression of the transgene in the NOD background. Indeed, in contrast to what is seen in FVBN-RIPN mice where close to 100% of insulin-expressing cells express the RIPN transgene [14], only 44±14% (mean ±95%CI; 58 islets from 6 five week-old NOD-RIPN mice) of the insulin positive cells were expressing the transgene (i.e. were HA-positive) (see Figure 1B for a representative example of this mosaicism). Despite this, islets isolated from NOD-RIPN mice were nevertheless significantly more resistant to inflammatory cytokine-induced apoptosis compared to islets isolated from NOD control mice (Figure 1C). This indicates that fragment N is functional in its ability to induce protective signals in beta cells of NOD mice.

**Table 1:** Pancreatic histological sections from mice of the indicated age stained with anti-insulin antibodies and with Hoechst 33342 were used to determine the indicated parameters (see material and methods).

Week 5	Parameter	NOD	NOD-RIPN
	Area of insulin positive cells (% of pancreas section)	0.61±0.26	0.89±0.12
	Single islet surface (µm^2^)	3868±1804	4471±996
	Number of insulin positive cells per islet	19±8	21±4
	Number of islets per section	29±7	37±12
	Area of a single insulin positive cell (µm^2^)	202±41	211±34

Week 10	Parameter	NOD	NOD-RIPN
	Area of insulin positive cells (% of pancreas section)	0.50±0.15	0.77±0.43
	Single islet surface (µm^2^)	4998±2122	4673±1667
	Number of insulin positive cells per islet	20±9	20±6
	Number of islets per section	33±9	34±11
	Area of a single insulin positive cell (µm^2^)	251±11	231±30

Week 16	Parameter	NOD	NOD-RIPN
	Area of insulin positive cells (% of pancreas section)	0.37±0.23	0.37±0.21
	Single islet surface (µm^2^)	4447±1966	4856±2281
	Number of insulin positive cells per islet	20±11	26±14
	Number of islets per section	17±11	19±14
	Area of a single insulin positive cell (µm^2^)	184±27	188±43

Six female mice per genotype and per age were used (one section per animal). The results are represented as mean±95% CI. There was no statistical significant difference between NOD and NOD-RIPN mice as assessed by Student t test for any of the tested parameter.

### Protective signaling induced by fragment N in beta cells

The anti-apoptotic kinase Akt is required for N to exert its protective functions both in beta cell lines and non-beta cells [8], [10], [19]. Figure 2A shows that this kinase is, as expected, constitutively activated in islets isolated from NOD-RIPN mice. While Akt has the ability to stimulate the NFκB transcription factor, it does not so in the presence of fragment N [10]. Moreover, the presence of fragment N in beta cells of FVB/N mice hampers NFκB stimulation by cytokines [14] and the same is observed in islets isolated from NOD-RIPN mice (Figure 2B). This might be important in the context of pancreatic beta cell survival as sustained activation of NFkB induces the death of beta cells [27]. Indeed, inhibition of NFkB specifically in beta cells *in vivo*, protects mice from developing multiple low-dose streptozotocin-induced diabetes [18]. To evaluate the potential beneficial effect of blocking NFκB when Akt is stimulated in insulin secreting cells, INS1 were transfected with a plasmid encoding a constitutive form of Akt (myr-Akt) with or without plasmids encoding either fragment N or a super-repressor of NFκB (a truncated version of IκBα that cannot be degraded and hence that chronically binds to and inhibits NFκB). Figure 2C shows that expression of myr-Akt stimulates NFkB activity in INS1 cells but this also induces their apoptosis. However, blocking NFkB activity with the NFkB super-repressor or with fragment N (Figure 2C, left panel) restores the viability of INS1 cells expressing myr-Akt (Figure 2C, right panel). This indicates that fragment N, through its capacity to inhibit NFkB, prevents beta cell death induced by an active form of Akt. However, Akt is required for fragment N to mediate its protective function in beta cells [19]. Therefore, in the case of beta cells, both activation of Akt and blockade of NFkB appear required for an efficient protection induced by fragment N.

### The effect of fragment N expression on type 1 diabetes development

To determine if the RIPN transgene could affect the auto-immune islet attack occurring in the NOD background, the extent of lymphocytic infiltration was scored (see material and methods) at 5, 10, and 16 weeks of age. Figure 3A shows a significant increase in lymphocyte infiltration in mice of both genotypes between 5 and 10 weeks of age. The lymphocyte infiltration however did not differ between NOD and NOD-RIPN mice. Additionally, the percentage of T cells (i.e. CD3-positive cells) in the infiltrated area did not differ between the two strains of mice (Figure 3B). The kinetics and mode of activation of the auto-immune attack on islets therefore does not seem to be affected by the presence of fragment N in beta cells of NOD mice.

To determine whether fragment N modulates the development of diabetes due to the NOD background, cohorts of male and female NOD and NOD-RIPN mice were followed for their glycaemia up to 44 weeks of age (Figure 4). During this period ∼90% of the females and ∼50% of the males, irrespectively of the presence of the RIPN transgene, developed diabetes. There was no statistical difference between the NOD and NOD-RIPN mice in terms of reduction over time of the number of mice with normo-glycaemia (non-fasted glycaemia below 10 mM; Figure 4A) or mice without overt diabetes (non-fasted glycaemia below 20 mM; Figure 4B). There was a clear trend in NOD-RIPN males, however, to maintain a glycaemia between 10 and 20 mM and, even more strikingly, to become overtly diabetic later than NOD controls (Figure 4A–B).

**Figure 5:**
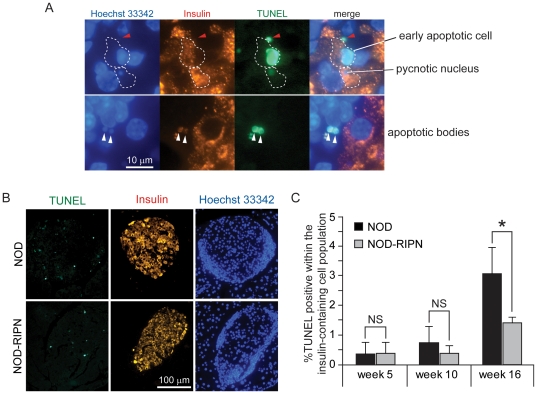
Apoptosis *in situ*. Mice at the indicated ages were killed and apoptosis of insulin-expressing cells on islet sections was determined by the TUNEL assay. **A.** Examples of early and late apoptotic cells as well as apoptotic bodies (white arrowheads). The structure indicated by the red arrowhead is not considered as an apoptotic cell because it does not contain DNA (i.e. not stained with the Hoechst dye). Such structures are not included in the quantitation shown in panel B. **B.** Representative images of whole islet sections from 16 week-old animals stained for insulin and DNA and subjected to TUNEL. **C.** Quantitation of TUNEL-positive cells within the insulin-containing cell population. Results are derived from 5–6 mice per age group and genotype.

Figure 1 showed that ∼40% of beta cells in the NOD-RIPN mice did not express the transgene, suggesting that half of the insulin-secreting cells in these mice will not benefit from the potential protection conferred by fragment N. As the auto-immune attack on islet cells does not seem to be affected by fragment N (see Figure 3), this could explain why the kinetics of appearance of a glycaemia over 10 mM was not significantly different between NOD and NOD-RIPN mice (Figure 4A). The stochastic nature of appearance of diabetes in the NOD model [20], combined with the partial penetrance of fragment N expression in NOD-RIPN mice, could explain the lack of significance in the development of overt diabetes between NOD and NOD-RIPN mice (Figure 4B). However, when taken individually, NOD-RIPN mice were found to remain in an overtly diabetes-free condition (i.e. non-fasted glycaemia below 20 mM) significantly longer than the NOD controls once their glycaemia exceeded 10 mM for the first time (Figure 4C–D). This was particularly striking in males. This protective effect of fragment N was also seen when apoptosis *in situ* was assessed in insulin-containing cells in 16 week-old mice (Figure 5).

## Discussion

In the present work, we provide evidence that fragment N expressed in ∼40% of the pancreatic beta cells of NOD mice significantly increases the time these mice can remain free of overt diabetes (>20 mM) once they have started to lose their ability to maintain a normo-glycaemia (i.e. <10 mM). Presumably, this protective effect would have been greater were fragment N expressed in more beta cells. The reason why the transgene encoding fragment N is expressed in virtually all beta cells of FVB/N mice [14] but less than half of the beta cells after the back-cross in the NOD background is unclear. There are however precedents for such observations. For example, the expression in beta cells of a transgene coding for the myristoylated active form of Akt was initially reported to be close to 100% [28] but became mosaic when it was further backcrossed into the C57BL/6 background [29].

T cell-mediated apoptosis of beta cells is the cause of development of type 1 diabetes [30]. In recently diagnosed type 1 diabetic patients, a residual population of beta cells appear to remain however and there is even evidence that there is on-going beta cell production in long-standing type 1 diabetes (discussed in reference [31]). While there is no information on the mechanisms that could potentially mediate beta cell replication or beta cell differentiation in adult human individuals, work performed in mice has revealed at least two independent ways that can lead to an increase in beta cell mass in rodents: replication from pre-existing beta cells [32], [33] and trans-differentiation from glucagon producing alpha cells [34]. In the context of immuno-suppression, it is interesting to note that proliferation of a pre-existing beta cell pool is inhibited by the non-steroid immuno-suppressant drugs used during islet transplantation [33], a very unfortunate side effects of these drugs that could block a potential capacity of the grafted islets to increase their number of beta cells.

Regardless of the underlying mechanisms that would allow a therapeutic treatment to favor an increase in beta cell mass in type 1 diabetic patients, raising the intrinsic resistance of beta cells to apoptosis should in principle provide an additional benefit to the treatment.

Apoptosis has been reported to occur before the lymphocytic infiltration in islets of NOD mice [35]. Fragment N through its ability to inhibit apoptosis could therefore have had a negative effect on the development of insulinitis that could explain its ability to delay diabetes development in the NOD background. However, NOD-RIPN mice experienced the same extent of immune infiltration as control NOD mice (Figure 3) and there was no difference in the rate of beta cell apoptosis before immune infiltration (Figure 5). Fragment N does not therefore affect the events that will induce the auto-immune attack on beta cells in NOD mice but it protects beta cells against the infiltrated immune cells.

Other anti-apoptotic proteins than fragment N have been tested in the NOD background for their ability to protect beta cells against apoptosis. CrmA is a viral protein of the serpin family that potently inhibits caspases [36]. When expressed in beta cells of NOD mice this protein led to a reduction in the spontaneous development of diabetes [37]. The use of CrmA as a protective device in beta cells would however be problematic because of the potential immune reaction that can be induced by a viral protein. Bcl-2 is the prototypic member of the Bcl-2 family of proteins [38]. It inhibits the activity of pro-apoptotic Bcl-2 family members and can prevent cell death of cells in response to a variety of stimuli. It has no effect however on the development of diabetes when expressed in the beta cells of NOD mice [39]. Fragment N is therefore a better beta-cell protector *in vivo* compared to Bcl-2, which is consistent with *in vitro* data obtained using the insulinoma cell lines betaTC-tet [19]. If combined with treatments aimed at reducing the strength of the autoimmunity, tools based on the ability of fragment N to protect beta cells could contribute to maintain an appropriate glucose control in patients who might otherwise become diabetic.
